# Efficient self-regulation of human functional states as a predictor of work success under long-term innovation stress

**DOI:** 10.1192/j.eurpsy.2022.1776

**Published:** 2022-09-01

**Authors:** A. Kuznetsova, M. Titova

**Affiliations:** Lomonosov Moscow State University, Department Of Psychology, Moscow, Russian Federation

**Keywords:** human functional state, effective self-regulation of functional states, tensed work conditions, work

## Abstract

**Introduction:**

Research data indicate the necessity of efficient human functional states’ (HFS) self-regulations for successful work execution, and not only for those professionals, who work under extreme work conditions; efficient HFS self-regulation is discussed as one of the key professional competences in socionomic jobs as well (Friedman, 2003; Landy & Conte, 2021). Moreover, ability of efficient HFS self-regulation could be viewed as a differentiative competence for separation of professionals with normal and high work achievements (Spencer L. & Spencer S., 1993).

**Objectives:**

The empirical study was targeted to investigate HFS self-regulation efficiency as a predictor of work success under long-term innovation stress.

**Methods:**

The longitudinal research was conducted in college teachers (n=50) during the period of organizational innovations. The empirical data were obtained by the diagnostic set of methods for self-assessment of attitudes towards innovations as a factor of long-term work strain, coping strategies and self-regulation means, chronic fatigue and burnout as the main consequences of long-term stress manifestations (Hobfoll, Dunahoo, Ben-Porath & Monnier, 1994; Leonova, 2012).

**Results:**

The significant diversity in the level of HFS self-regulation efficiency in teachers with different work success has been found (p<0,05). Teachers with the highest level of work results have a well-developed system of self-regulation means, which allows them to cope with innovation stress.

**Conclusions:**

The empirical data revealed patterns of effective self-regulation means, which are adequate to situational requirement, professional norms and rules of organizational behavior. Obtained results formed the basis for the elaboration of training course for HFS self-regulation skills development.

**Disclosure:**

No significant relationships.

